# Bulk regional viral injection in neonatal mice enables structural and functional interrogation of defined neuronal populations throughout targeted brain areas

**DOI:** 10.3389/fncir.2015.00072

**Published:** 2015-11-05

**Authors:** Claire E. J. Cheetham, Bryce D. Grier, Leonardo Belluscio

**Affiliations:** ^1^National Institute of Neurological Disorders and StrokeBethesda, MD, USA; ^2^Department of Biological Sciences, Carnegie Mellon UniversityPittsburgh, PA, USA

**Keywords:** adeno-associated virus, bulk labeling, neural circuit, olfactory bulb, tufted cell

## Abstract

The ability to label and manipulate specific cell types is central to understanding the structure and function of neuronal circuits. Here, we have developed a simple, affordable strategy for labeling of genetically defined populations of neurons throughout a targeted brain region: Bulk Regional Viral Injection (BReVI). Our strategy involves a large volume adeno-associated virus (AAV) injection in the targeted brain region of neonatal Cre driver mice. Using the mouse olfactory bulb (OB) as a model system, we tested the ability of BReVI to broadly and selectively label tufted cells, one of the two principal neuron populations of the OB, in CCK-IRES-Cre mice. BReVI resulted in labeling of neurons throughout the injected OB, with no spatial bias toward the injection site and no evidence of damage. The specificity of BReVI labeling was strikingly similar to that seen previously using immunohistochemical staining for cholecystokinin (CCK), an established tufted cell marker. Hence, the CCK-IRES-Cre line in combination with BReVI can provide an important tool for targeting and manipulation of OB tufted cells. We also found robust Cre-dependent reporter expression within three days of BReVI, which enabled us to assess developmental changes in the number and laminar distribution of OB tufted cells during the first three postnatal weeks. Furthermore, we demonstrate that BReVI permits structural and functional imaging *in vivo*, and can be combined with transgenic strategies to facilitate multi-color labeling of neuronal circuit components. BReVI is broadly applicable to different Cre driver lines and can be used to regionally manipulate genetically defined populations of neurons in any accessible brain region.

## Introduction

The recent development of a large repertoire of Cre driver lines (Gong et al., 2007; Taniguchi et al., 2011) has greatly facilitated cell type-specific labeling, monitoring and manipulation of neuronal circuits. Reporter gene expression can be achieved either by breeding these Cre driver mice with Cre reporter lines, e.g., (Madisen et al., 2010, 2012; Chen et al., 2013), or instead by injecting Cre driver mice with recombinant Cre-dependent reporter viruses. Viral transduction provides both spatial and temporal control of gene expression, and benefits from higher expression levels of reporters (Atasoy et al., 2008; Kuhlman and Huang, 2008). In particular, Cre-dependent recombinant AAVs have become a widespread tool for cell type-specific reporter gene expression (Betley and Sternson, 2011). AAV is typically delivered by stereotaxic injection in juvenile or adult mice, either at a single site or at multiple sites along a single track. While this strategy has great utility, it precludes studies of early postnatal development of neuronal circuits, and expression is restricted to a relatively small number of neurons around the injection site(s).

We assessed the ability of **B**ulk **Re**gional **V**iral **I**njection (BReVI) to achieve population labeling throughout the targeted brain region of Cre driver mice, beginning in early postnatal life. We tested BReVI primarily in the olfactory bulb (OB), the initial site of odor processing in the mammalian brain. Here, the principal neurons of the OB, the mitral and tufted cells, process odor information received from olfactory sensory neurons in the nose before transmitting it to olfactory cortex. While often considered to be functionally equivalent, mitral and tufted cells in fact transmit temporally distinct signals to spatially segregated areas of olfactory cortex (Nagayama et al., 2014). Specifically, mitral and tufted cells lock to opposing phases of the sniff cycle, with tufted cells responding with shorter onset latencies and higher firing rates to odor stimulation (Nagayama et al., 2004; Griff et al., 2008; Fukunaga et al., 2012; Igarashi et al., 2012). Anatomically, individual mitral and tufted cells target largely non-overlapping areas within the anterior olfactory nucleus, olfactory tubercle and anterior piriform cortex, while mitral cells also project to the posterior piriform cortex, entorhinal cortex, and cortical amygdaloid nuclei (Haberly and Price, 1977; Scott, 1981; Nagayama et al., 2010; Igarashi et al., 2012). Furthermore, tufted cell axons also form intrabulbar projections, which target the isofunctional odor column on the opposite side of the OB (Schoenfeld et al., 1985; Liu and Shipley, 1994; Belluscio et al., 2002). While mitral and tufted cells can be distinguished based on their soma location, dendritic morphology and biophysical properties (Haberly and Price, 1977; Scott, 1981; Macrides and Schneider, 1982; Nagayama et al., 2010; Igarashi et al., 2012; Burton and Urban, 2014; Nagayama, 2014), there is a need for genetic markers that can be used to selectively label and manipulate tufted vs. mitral cells *in vivo*. The neuropeptide cholecystokinin (CCK) is expressed in tufted but not mitral cells (Seroogy et al., 1985), making the CCK-IRES-Cre line (Taniguchi et al., 2011) an excellent candidate to enable reporter expression selectively in tufted cells.

Here, we show that population labeling of tufted cells throughout the OB of CCK-IRES-Cre mice can be achieved using BReVI. Reporter expression showed no spatial bias toward the injection site, and was evident within 3 days of injection. This enabled us to track the number and laminar position of tufted cell sub-classes during early postnatal life, and highlighted a population of CCK+ neurons residing in the mitral cell layer (MCL). We also demonstrate that BReVI can be used for both structural and functional imaging *in vivo*, and to achieve cell type-specific expression in other Cre lines and other accessible brain regions.

## Methods

### Experimental Animals

All animal procedures conformed to National Institutes of Health guidelines and were approved by the National Institute of Neurological Disorders and Stroke Institutional Animal Care and Use Committee. Mouse lines were obtained from The Jackson Laboratory and bred in-house. Experimental mice were homozygous CCK-IRES-Cre (#12706) (Taniguchi et al., 2011), hemizygous TH-Cre (#8601) (Savitt, 2005), TH-Cre^+/-^;ROSA-CAG-LSL-tdTomato^+/-^ [generated by breeding lines #8601 and #7909 (Madisen et al., 2010)] or OMP-GFP^+/-^;Thy1-YFP-G^+/-^;TH-Cre^+/-^ [generated by breeding lines #6667, #14130, and #8601 (Feng et al., 2000; Potter et al., 2001)]. Mice were maintained on a 12 h light/dark cycle with food and water *ad libitum*. PCR-based genotyping protocols were as described on the Jackson Laboratory website. Mice expressing both Thy1-YFP-G and OMP-GFP were identified by fluorescence screening using a stereomicroscope (Leica) on postnatal day (P)2. A total of 30 mice were used in this study.

### Bulk Regional AAV Injection in Neonatal Mice

AAV was obtained from Penn Vector Core. Viruses used were AAV1.CAG.Flex.tdTomato.WPRE.bGH (AAV1-Flex-tdTomato; AV-1-ALL864), AAV1.CAG.Flex.GCaMP6f.WPRE (AAV1-Flex-GCaMP6f; AV-1-PV2816), and AAV1.CAG.Flex.eGFP.WPRE.bGH (AAV1-Flex-GFP; AV-1-ALL854). P0–P3 mouse pups were cryo-anesthetized (Phifer and Terry, 1986) in an ice-cold chamber, taking care to avoid directly contacting the pups with ice. Prior to injection, each pup was positioned dorsal side up, with its head facing to the right (for injection by a right-handed experimenter) and secured with an adhesive bandage across the upper body. AAV injections were made using a custom-made injector, consisting of a 1ml luer-lock syringe connected via flexible tubing to a pulled glass micropipette with a 30–50 μm-diameter beveled tip. Gentle negative pressure on the injector syringe was used to load 1 μl of AAV suspension into the micropipette. For injection, the head was gripped gently and the tip of the micropipette was inserted carefully through the skin and cranium. Gentle positive pressure was applied on the injector syringe to eject 1 μl of AAV, taking care not to inject air into the brain. Anatomical landmarks used to guide injections, including the inferior cerebral vein and superior sagittal sinus, were visible through the skin up to and including P3. Frontal cortex injections were made just rostral to the inferior cerebral vein, close to the midline. Pups recovered on a heat pad and were returned to their home cage once awake and re-warmed. Survival rate for this procedure was 100%, and injection of a litter of 10–12 pups took ∼30 min, including recovery time.

### Perfusion and Tissue Processing

Mice were deeply anesthetized with 200 mg/kg ketamine and 20 mg/kg xylazine (Vedco) and transcardially perfused with ice-cold PBS followed by 4% PFA. Brains were dissected out and post-fixed overnight in 4% PFA at 4°C, cryopreserved in 30% sucrose in PBS for 24 h at 4°C, embedded in 10% gelatin, fixed/cryopreserved in 15% sucrose/2% PFA in PBS overnight, and flash frozen in 2-methyl butane on dry ice. Coronal sections were cut using a cryostat (Leica Microsystems CM3050S, or Microm) at 25 or 40 μm. Sections were mounted directly in Vectashield containing DAPI (Vector Labs) or ProLong Diamond containing DAPI (Life Technologies), or stored at -80°C.

### Immunohistochemistry

Free-floating sections were blocked in 2% normal donkey serum/0.1% Triton X-100 for 1 h, then incubated with rabbit anti-Iba1 primary antibody (Wako, 1:2000) and then anti-rabbit-AF647 secondary antibody (Molecular Probes, 1:600) in 2% normal donkey serum/0.05% Tween 20 for 1h each at RT before mounting in Vectashield or ProLong Diamond.

### Confocal Imaging

Images of CCK-IRES-Cre tissue were acquired with a Nikon A1R confocal microscope equipped with Plan Fluor 10x/0.3NA air and Apo 60x/1.4NA oil immersion objectives. Images of TH-Cre and OMP-GFP^+/-^;Thy1-YFP-G^+/-^;TH-Cre^+/-^ tissue were acquired using a Leica TCS SP5 equipped with HCX PLAN FLUOTAR 10x/0.3NA air and HCX PL APO 40x/1.25NA oil immersion objectives and spectral detectors. GFP and YFP fluorescence were collected sequentially and unmixed using LASAF software (Leica).

### Cell Counts

Laminar position was assigned according to the following criteria: cells in the glomerular layer (GL) were juxtaglomerular, i.e., encircled a glomerulus; the superficial EPL (sEPL) was the superficial half of the EPL, containing dense tdTomato-labeled lateral dendrites; the deep EPL (dEPL) was the deep half of the EPL extending to the border of the MCL; the MCL was defined as the dense band of somata identified by DAPI staining; the internal plexiform layer (IPL) was immediately deep to the MCL with sparse nuclei and tdTomato-labeled axons in older mice; and the granule cell layer (GCL) commenced where nuclei became more densely packed, deep to the IPL. For the data in **Figure 4**, tdTomato-labeled neurons in CCK-IRES-Cre mice were counted in 40x confocal z-stacks. Iba1-stained microglia and labeled granule cells in TH-Cre mice were counted in 10x confocal images. For the comparison of labeled neuron density with CCK-IRES-Cre;ROSA-CAG-LSL-tdTomato mice, tdTomato-expressing neurons were counted in 10x confocal images of 25 μm sections from P14 CCK-IRES-Cre mice injected with AAV1-FLEX-tdTomato at P0. Counts for P14 CCK-IRES-Cre;ROSA-CAG-LSL-tdTomato mice were made from images in the Allen Developing Mouse Brain Atlas (Allen Developing Mouse Brain Atlas, 2015). Linear densities were calculated using the circumference of the GL/EPL and EPL/MCL borders, respectively.

### *In Vivo* 2-photon Imaging

Mice were anesthetized using isoflurane (4% induction, 1.5–2% maintenance in O_2_) and ∼1 mm diameter cranial windows were implanted over the right OB as described (Holtmaat et al., 2009). Lightly anesthetized mice (0.5 mg/kg s.c. dexmedetomidine plus 1% sevoflurane in O_2_) were imaged using a Leica TCS SP5 microscope using a HCX APO 20x/1.0NA water-immersion objective lens and a Chameleon Vision II laser (Coherent) mode-locked at 910 nm. tdTom emission was collected at >600 nm and GCaMP6f emission at 500–550 nm.

To image neuronal structure in CCK-IRES-Cre mice, z-stacks with voxel size 0.42 × 0.42 × 2 μm or 0.12 × 0.12 × 1 μm were acquired. Images were collected using Leica non-descanned HyD detectors, and excitation power was ramped up with increasing depth from the surface, enabling detection of fluorescence signals at depths >400 μm. For time-lapse imaging in TH-Cre mice, z-stacks with voxel size 0.12 × 0.12 × 1 μm were acquired at 1 h intervals for 3 h, with excitation power constant throughout the stack and across time points. Images were collected with Leica non-descanned PMT detectors.

For GCaMP6f imaging, ethyl butyrate (EB) was dissolved in mineral oil (1% volume/volume) and delivered to the anesthetic air stream using a PV-820 Pneumatic PicoPump (World Precision Instruments). The timing of odorant delivery and image acquisition were controlled using LASAF software (Leica). Each imaging trial lasted 20 s, consisting of 3 s of baseline, 3 s of odor presentation, and 14 s after stimulus termination. Images (0.24 μm/pixel, 512 × 512) were collected at three frames/s and there was a 90 s interval between trials.

### Statistics

Data were compared using paired *t*-tests (microglial density), one-way ANOVA (soma diameter) or two-way ANOVA (cell counts in CCK-IRES-Cre mice injected with AAV1-Flex-tdTomato). All statistical tests were performed using Prism 6 (GraphPad).

## Results

### BReVI Labels Tufted Cells throughout the OB

We assessed the ability of BReVI, which comprises a single large-volume AAV injection in the neonatal brain, to label a genetically defined population of neurons throughout the targeted brain region. We tested BReVI in the mouse OB, which has a radial, laminated structure that makes it ideally suited to assess the spatial extent of AAV-mediated labeling in three dimensions. We focused on labeling of tufted cells, one of the two principal neuron populations of the OB, which were genetically targeted using the CCK-IRES-Cre knock-in line (Taniguchi et al., 2011) and AAV encoding a fluorescent Cre reporter.

**FIGURE 1:**
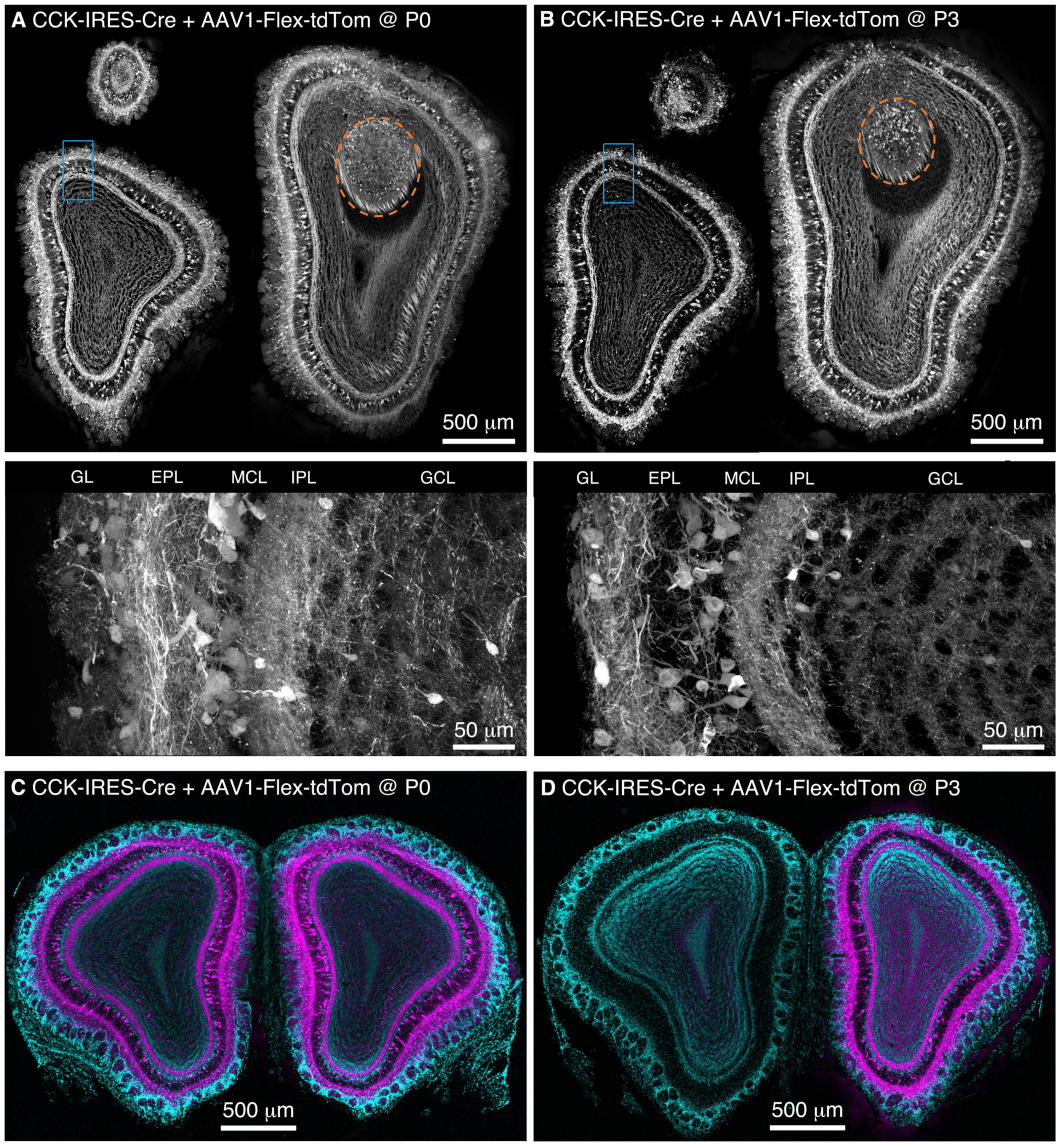
**Population labeling of CCK+ neurons throughout the OB following Bulk Regional Viral Injection (BReVI) in neonatal mice. (A,B)** Coronal sections at anterior, central, and posterior positions along the rostrocaudal axis showing labeling of OB neurons in P21 CCK-IRES-Cre mice injected with AAV1-Flex-tdTomato **(A)** at P0 and **(B)** P3. Orange dashed lines enclose the AOB. Higher magnification images of blue boxed regions are shown below. GL, glomerular layer. EPL, external plexiform layer. MCL, mitral cell layer. IPL, internal plexiform layer. GCL, granule cell layer. **(C,D)** Coronal sections showing the injected (right) and uninjected (left) OBs of P21 CCK-IRES-Cre mice that received OB injections of AAV1-Flex-tdTomato at **(C)** P0 or **(D)** P3.

We performed freehand injections of 1 μl of AAV1-Flex-tdTomato near the dorsal surface of the right OB in P0 CCK-IRES-Cre mice. Three weeks post-injection, we observed tdTomato-expressing neurons, predominantly concentrated in the deep GL, EPL, and MCL in OB sections (**Figure 1A**). Labeled axons formed a dense band in the IPL, and were more sparsely distributed in the GCL (**Figure 1A**), while labeled lateral dendrites were in the superficial half of the EPL, characteristic of tufted cells (Mori et al., 1983; Igarashi et al., 2012). This pattern of labeling closely matches that reported previously using anti-CCK immunohistochemistry and *in situ* hybridization (ISH) in the rodent OB (Seroogy et al., 1985; Liu and Shipley, 1994; Lein et al., 2006; Marks et al., 2006; Allen Mouse Brain Atlas, 2015). Importantly, labeling density and intensity were uniform along the dorsal-ventral, medial-lateral, and rostro-caudal axes of the OB. Neurons in the accessory olfactory bulb (AOB), a region that is difficult to target directly for injection, were also labeled (**Figure 1A**). To estimate the proportion of CCK-expressing neurons that were transduced by AAV, we compared the density of labeled neurons in mice injected at P0 with AAV1-FLEX-tdTomato (156 ± 4 cells/mm at the GL/EPL border, 63 ± 1 cells/mm at the EPL/MCL border, *n* = 2) with that in age-matched CCK-IRES-Cre;ROSA-CAG-LSL-tdTomato mice (171 ± 1 cells/mm at the GL/EPL border, 72 ± 8 cells/mm at the EPL/MCL border, *n* = 2). Overall, approximately 90% of the number of neurons labeled in the Cre reporter line cross were labeled in mice injected with AAV at P0, indicating that the majority of Cre-expressing neurons were transduced with AAV.

We then repeated our AAV1-Flex-tdTomato injections in P3 CCK-IRES-Cre mice, the maximum age at which anatomical landmarks remained visible through the skin. Again, tdTomato-expressing neurons were present throughout the OB, with no bias towards the dorsally located injection site (**Figure 1B**). The laminar distribution of labeled neurons was very similar to that seen following P0 injection. Interestingly, we noted that in P0-injected mice, tufted cells throughout the OB contralateral to the injection site were also brightly labeled (**Figure 1C**). In marked contrast, in P3-injected mice, there was little or no labeling of tufted cells in the contralateral (uninjected) OB (**Figure 1D**). Some labeling of centrifugal fibers in the GCL of the contralateral OB was seen following P3 injection, which presumably arises from retrograde infection of bilaterally projecting CCK-expressing neurons in the anterior olfactory nucleus (Davis and Macrides, 1981; Rothermel et al., 2013). The robust difference in contralateral labeling between P0 and P3 injections suggests that the timing of neonatal AAV injection could be used to adjust the extent of contralateral OB labeling for different applications. The reasons for this developmental change are unclear, but the ‘all or none’ nature of contralateral OB tufted cell labeling suggests the formation of an anatomical barrier to diffusion of AAV between OBs, perhaps as a result of glial maturation.

**FIGURE 2:**
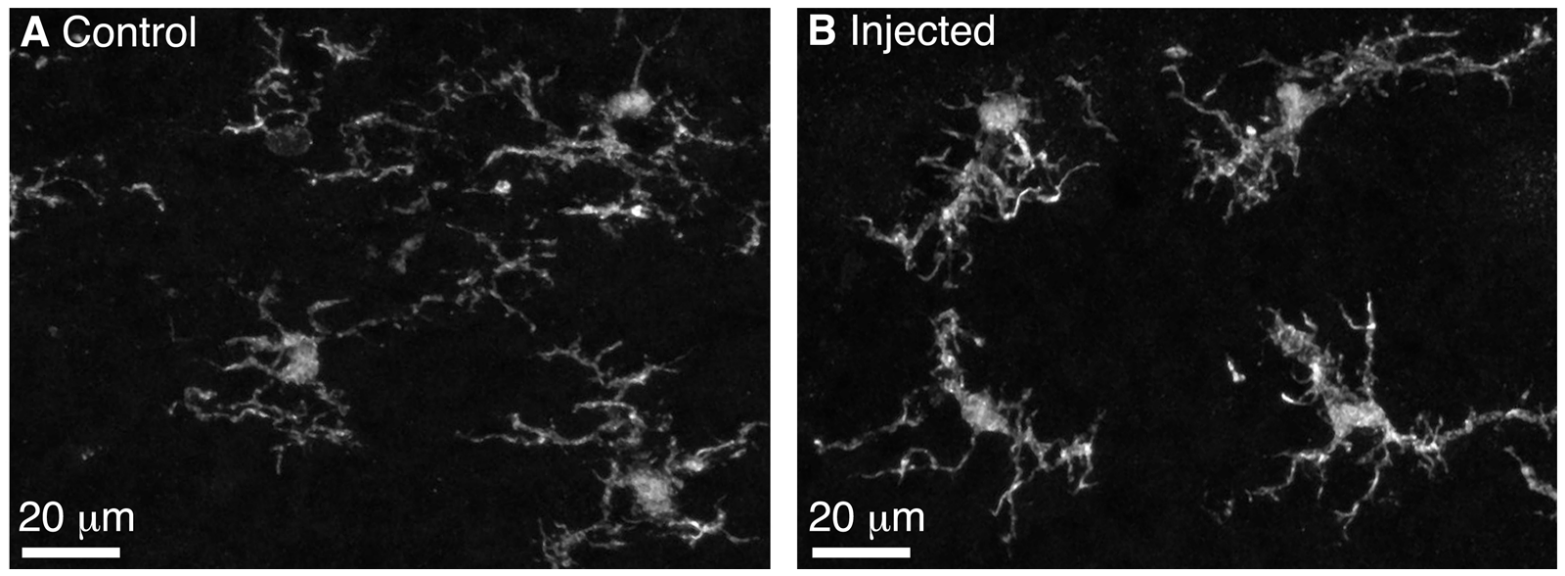
**Microglia in the control and injected OBs exhibit ramified morphology 5 days post-Bulk Regional Viral Injection. (A)** Maximum intensity projection (MIP) of confocal z-stack showing Iba1-stained microglia in the control OB contralateral to the injection site. **(B)** MIP of confocal z-stack showing Iba1-stained microglia in the injected OB. Note the ramified morphology typical of non-activated microglia.

We performed BReVI in a total of 25 neonatal CCK-IRES-Cre mice, with a 100% survival rate and no need to foster pups post-injection. We also examined serial cryostat sections through the OBs of these mice, and found no evidence of either spatial bias in tufted cell labeling within the OB, or of mechanical tissue damage to the OB resulting from the injection. Furthermore, the density of Iba1-labeled microglia was similar in the injected (57 ± 15 per mm^2^) and control contralateral OBs (65 ± 14 per mm^2^, *P* = 0.29, paired *t*-test). Microglia had similar ramified morphologies in both OBs (**Figure 2**), suggesting that AAV injection did not result in microglial activation. Therefore, we concluded that BReVI provides a reliable method for labeling of genetically defined populations of neurons throughout the mouse OB.

### Strong Expression of Fluorescent Cre Reporters within 3 Days of Neonatal AAV Injection

BReVI could enable analysis of the postnatal development of neuronal circuits. A potential barrier to this might be the relatively slow expression onset of AAV-transduced reporters (Coura and Nardi, 2007; Day et al., 2014). However, recent studies suggest that AAV-mediated reporter expression may in fact begin much earlier, within a few days of infection (Shevtsova et al., 2004; Kim et al., 2013). To clarify this issue, we injected AAV1-Flex-tdTomato in the right OB of CCK-IRES-Cre mice at P0, and assessed tdTomato expression 3 – 21 days later.

**FIGURE 3:**
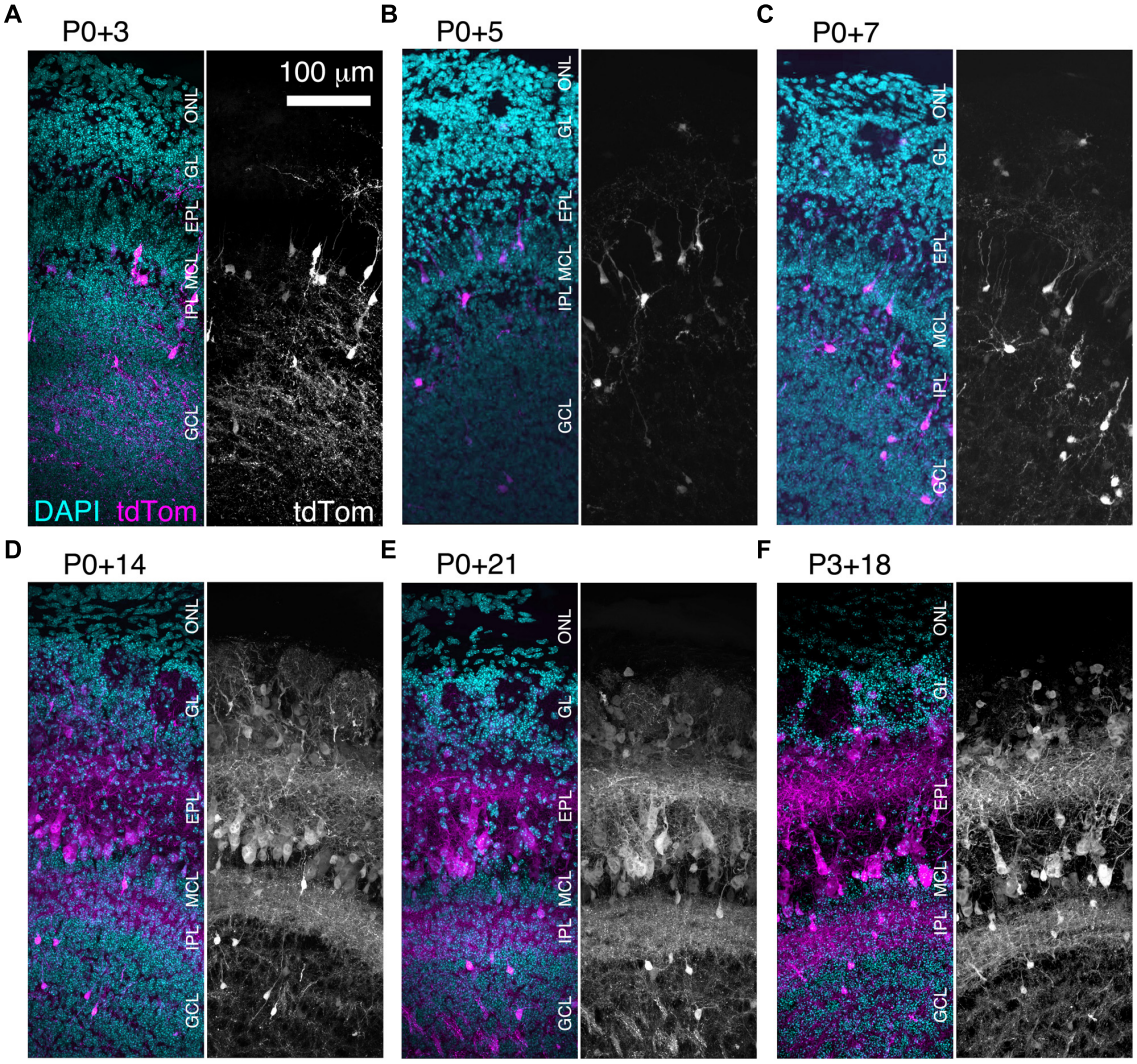
**Rapid expression onset of AAV-driven reporters enables developmental analyses. (A–E)** MIPs of confocal z-stacks of the lateral surface of the OB of CCK-IRES-Cre mice injected with AAV1-Flex-tdTomato at P0 and perfused at **(A)** P3, **(B)** P5, **(C)** P7, **(D)** P14, or **(E)** P21. **(F)** As **(A–E)** but mice were injected at P3 and perfused at P21. All images are of coronal sections centrally located along the rostrocaudal axis of the OB. ONL, olfactory nerve layer.

**FIGURE 4:**
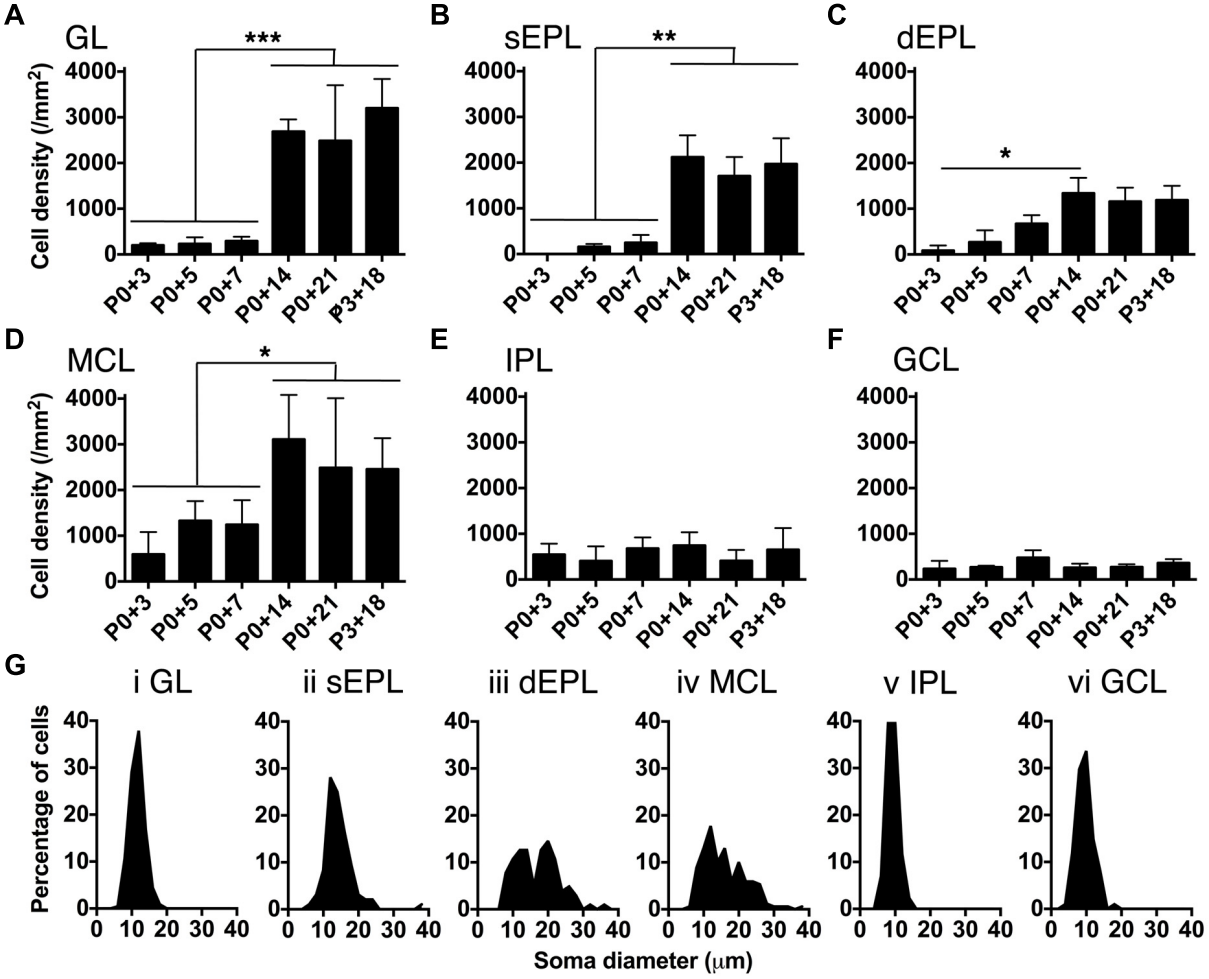
**Developmental changes in density of CCK-tdTomato neurons during early postnatal life. (A–F)** Density of tdTomato-expressing neurons in different OB layers from P3 to P21. *P* < 0.001 for effect of layer and effect of age, 2-way ANOVA; ^∗∗∗^*P* < 0.001, ^∗∗^*P* < 0.01, ^∗^*P* < 0.05 between age groups (Sidak multiple comparisons test). Note that brackets in **(A–D)** indicate significantly higher cell density in P14/P21/P3+18 groups than in P3/P5/P7 groups. **(G)** Soma diameter of tdTomato-expressing neurons in different OB layers at P21.

We found that brightly labeled tdTomato-expressing neurons were present throughout the OB as early as three days post-injection (**Figure 3A**), although their numbers and laminar positions differed from those of tdTomato-labeled neurons in older mice (**Figures 3B–F**). Therefore, we quantified the density of tdTomato-labeled neurons in different layers of the OB during the first three weeks of postnatal life (**Figures 3** and **4A–F**). In both the GL and sEPL, the density of tdTomato-labeled neurons, which correspond to external/superficial and middle tufted cells, increased dramatically between P7 and P14 (**Figures 3C,D** and **4A,B**). In the dEPL, the location of internal tufted cells, tdTomato-labeled cell density increased gradually from P3 to P14, and then stabilized (**Figure 4C**). This more gradual developmental change meant that differences between age groups mostly did not reach statistical significance (**Figure 4C**). Interestingly, there was also a high density of tdTomato-labeled neurons in the MCL in 2–3 week-old mice (**Figures 3D–F** and **D**). Many of these MCL-residing neurons had large somata (**Figure 4G**), which in combination with the lack of labeled secondary dendrites in the dEPL suggests that these may be type II mitral cells (Orona et al., 1984; Mouradian and Scott, 1988; Nagayama et al., 2014). Lateral dendrites in the sEPL and intrabulbar-projecting axons in the IPL were sparse at P7, but densely labeled by P14 (**Figures 3C,D**). Finally, scattered tdTomato-labeled neurons in the IPL and GCL were present at all ages (**Figures 3A–F** and **4E,F** ). These cells had small somata (**Figure 4G**) and hence are likely to be granule cells or deep short-axon cells that transiently expressed CCK during their development. We did not observe any differences in the density or laminar distribution of tdTomato-labeled neurons in 3-week-old mice injected at P0 vs. P3 (**Figures 3E,F** and **4A–F**). Overall, we concluded that BReVI-mediated labeling in CCK-IRES-Cre mice enables developmental analysis of OB tufted cells.

### Bright BReVI-mediated Fluorescent Protein Expression Enables *In Vivo* 2-photon Imaging of Neuronal Structure

**FIGURE 5:**
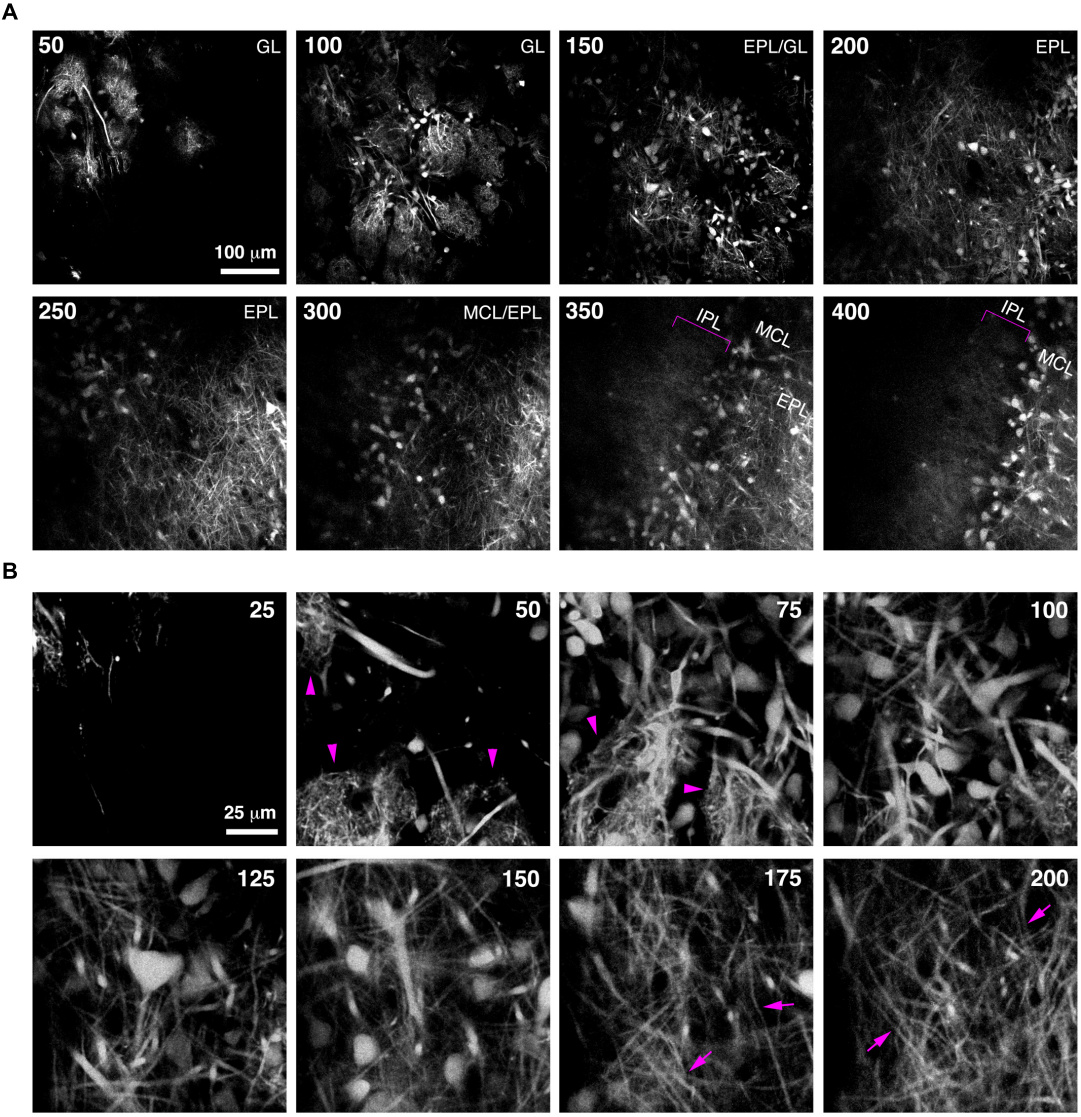
**Bulk Regional Viral Injection permits *in vivo* structural imaging of OB tufted cells. (A)** Single optical sections from a 2-photon z-stack of the dorsal surface of the OB of a 6 week-old CCK-IRES-Cre mouse injected with AAV1-tdTomato at P0. Numbers indicate depth in μm. Magenta brackets indicate intrabulbar-projecting tufted cell axons in the IPL at depths of 350–400 μm. **(B)** Same as A but different region of interest with higher magnification, illustrating tdTomato-expressing external and superficial tufted cells and lateral dendrites in the EPL. Magenta arrowheads indicate apical tufts at 50–75 μm depth, and magenta arrows indicate lateral dendrites at 175–200 μm depth.

Imaging of fine neuronal structures deep in the living brain requires strong expression of fluorescent proteins (Kuhlman and Huang, 2008). To assess whether BReVI results in expression levels sufficient to permit imaging in the adult OB, we injected CCK-IRES-Cre mice with AAV1-Flex-tdTomato and implanted cranial windows over the right (injected) OB six weeks post-injection. We then acquired 2-photon z-stacks spanning 400 μm in depth, from the GL down to the IPL. This enabled us to visualize large numbers of external/superficial, middle and internal tufted cells and their dendrites, as well as intrabulbar-projecting axons in the IPL at ∼350 μm in depth (**Figure 5A**, brackets). At higher magnification, the structure of apical dendritic tufts in the GL (**Figure 5B**, 50–75 μm depth; arrowheads) and lateral dendrites in the EPL (**Figure 5B**, 175–200 μm depth; arrows) can clearly be visualized. Hence, BReVI is a useful technique to enable structural analysis of genetically defined neurons *in vivo*.

### *In vivo* 2-photon GCaMP6f Imaging of Odor-evoked Responses in OB Tufted Cells Following Targeted Neonatal AAV Injection

**FIGURE 6:**
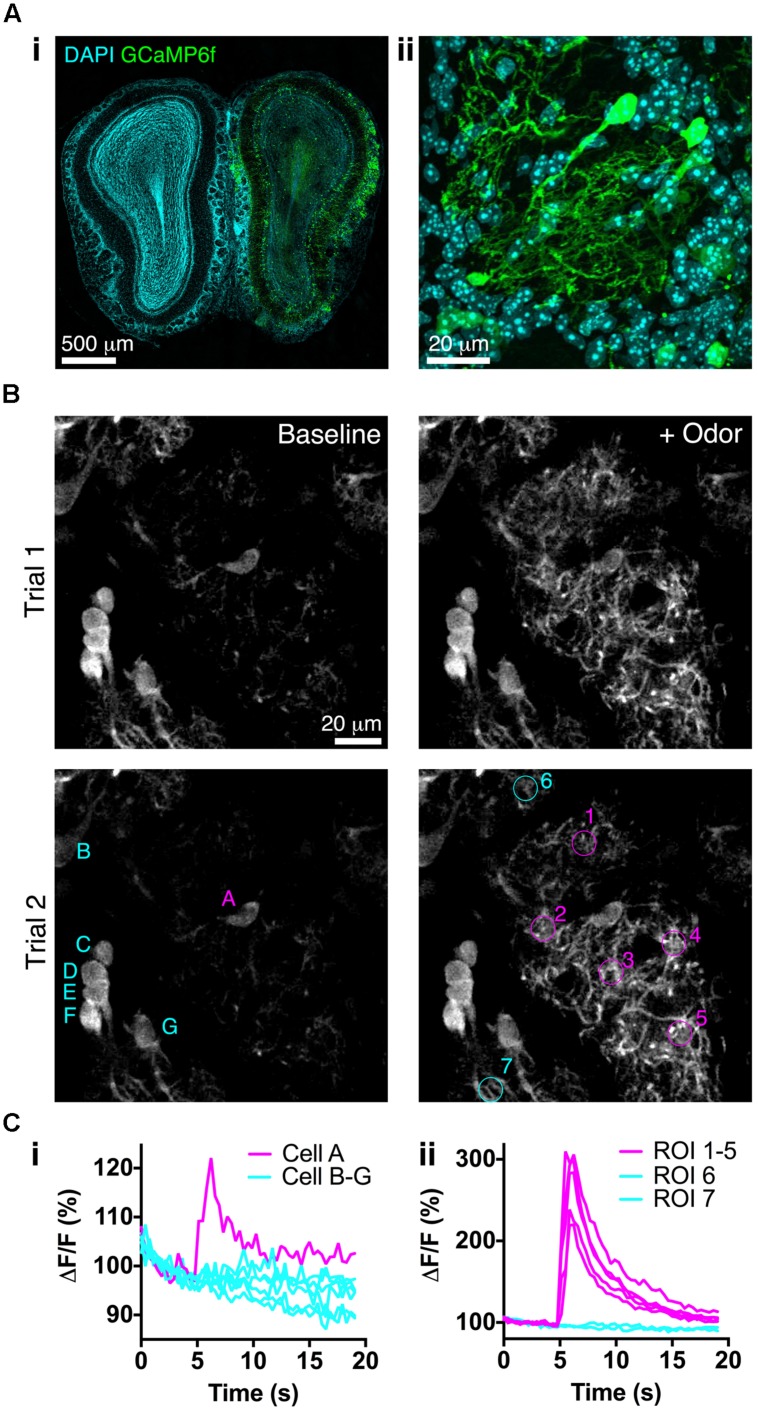
**Bulk Regional Viral Injection permits *in vivo* calcium imaging of OB tufted cells. (A) (i)** Coronal OB section from a P25 CCK-IRES-Cre mouse injected with AAV1-Flex-GCaMP6f at P3. Note low resting fluorescence of GCaMP6f. **(ii)** Single optical section showing GCaMP6f-labeled tufted cells. **(B)** *In vivo* 2-photon images showing responses to odor stimulation (1% EB, 3 s) in two separate trials at 90 s intervals. Overlaid letters and numbers correspond to traces in C. **(Ci)** Normalized ΔF/F for tufted cells marked in B lower left panel. Cell A is located at the periphery of the EB-responsive glomerulus. **(Cii)** Normalized ΔF/F for tufted cell dendrites in ROIs marked in B lower right panel. ROIs 1–5 correspond to the EB-responsive glomerulus; ROIs 6–7 correspond to adjacent unresponsive glomeruli.

We next assessed whether BReVI drives expression of a functional reporter sufficiently strongly to detect sensory responses *in vivo*. We injected P3 CCK-IRES-Cre mice with AAV1-Flex-GCaMP6f. Three weeks later, GCaMP6f fluorescence was detectable in a subset of tufted cells (**Figure 6A**). Many tufted cells in fixed tissue did not exhibit GCaMP6f fluorescence, consistent with the low resting fluorescence of GCaMP6 variants (Chen et al., 2013). We then implanted cranial windows over the right (injected) OB at P25, and performed *in vivo* 2-photon imaging in the GL. Again, resting fluorescence was low, primarily being evident in external tufted cell bodies (**Figure 6B**, left). In preliminary odor stimulation trials, we identified a glomerulus that exhibited robust responses to odor stimulation with 1% EB, and was surrounded by other glomeruli that did not respond to this odor (**Figure 6B**, right). We then quantified responses of both external tufted cells, and tufted cell dendrites, to EB stimulation. The external tufted cell located at the periphery of the EB-responsive glomerulus exhibited a significant response to EB stimulation, whereas other more distant external tufted cells showed no change in fluorescence during odor stimulation (**Figure 6Ci**). All regions of interest (ROIs) within the EB-responsive glomerulus showed robust, large magnitude fluorescence increases in response to EB stimulation, whereas ROIs in neighboring glomeruli showed no response (**Figure 6Cii**). Notably, ΔF/F was much greater for tufted cell dendrites than for the responsive external tufted cell (**Figure 6C**). Overall, these data demonstrate that BReVI-mediated labeling with GCaMP6f enables functional imaging of neuronal responses *in vivo*.

### BReVI Labels Dopaminergic Neurons Throughout the OB of TH-Cre Mice

**FIGURE 7:**
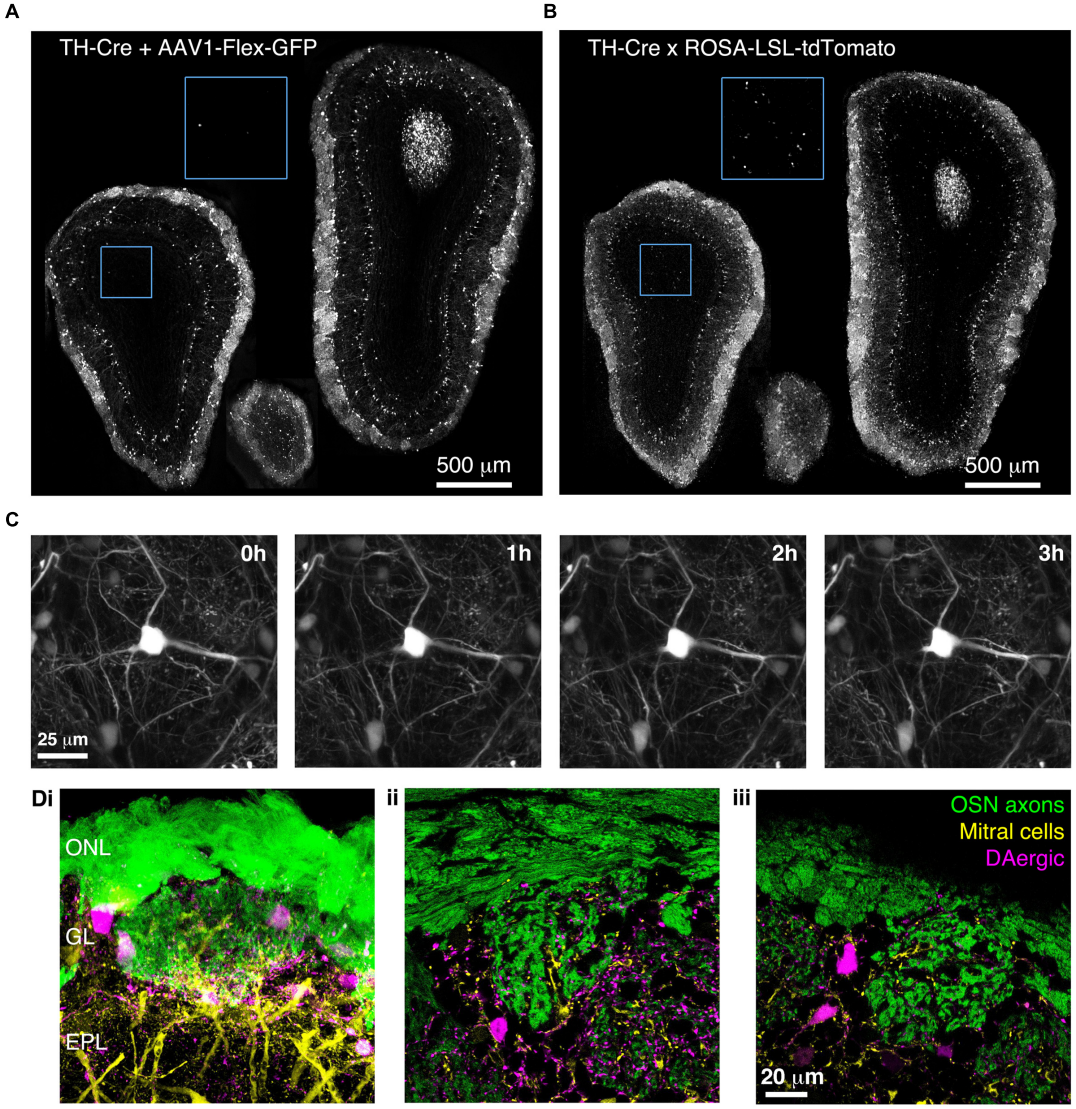
**Bulk Regional Viral Injection-mediated labeling of dopaminergic neurons throughout the OB of TH-Cre mice. (A)** Coronal sections at anterior, central and posterior positions along the rostrocaudal axis showing labeling of OB neurons in a P28 TH-Cre mouse injected with AAV1-Flex-GFP at P0. Blue boxed region illustrating density of labeled neurons in the GCL is shown at 2x higher magnification with increased contrast. **(B)** As A but for a P28 TH-Cre;ROSA-CAG-LSL-tdTomato mouse. **(C)** Time lapse 2-photon images (1 h intervals) of the dorsal surface of the OB of a P42 TH-Cre mouse injected with AAV1-Flex-GFP at P2. **(D)** Confocal images from a P56 OMP-GFP^+/-^;Thy1-YFP-G^+/-^;TH-Cre^+/-^ mouse injected with AAV1-Flex-tdTomato at P2, showing YFP labeling of mitral cell dendrites, GFP labeling of olfactory sensory neuron axons and tdTomato labeling of DAergic neurons. **(i)** MIP of confocal z-stack of the dorsal surface of the OB. **(ii, iii)** Single optical sections showing the medial and dorsal surfaces, respectively.

To confirm that BReVI can also be used to label other neuronal types in the OB, we performed P0 injections of AAV1-Flex-GFP in TH-Cre mice to label dopaminergic neurons, one of the regenerating populations of OB interneurons (Batista-Brito et al., 2008). We observed bright labeling of juxtaglomerular dopaminergic neurons and their processes in the GL 3 weeks post-injection (**Figure 7A**). Again, labeling was evident throughout the extent of the OB, and showed no spatial bias toward the dorsally located injection site (**Figure 7A**). Consistent with fluorescent reporter expression in TH-Cre;flox-reporter mice, some small neurons in the MCL, whose dendrites terminated in the EPL, were also brightly labeled (**Figure 7B**; Lindeberg et al., 2004; Adam and Mizrahi, 2011). Notably, although we also observed sparse labeling of neurons in the GCL in AAV-transduced TH-Cre mice (12 ± 7 cell per mm^2^; **Figure 7A**, blue boxed region), their density was much lower than in TH-Cre;ROSA-CAG-LSL-tdTomato mice (72 ± 20 per mm^2^, *P* < 0.001, *t*-test; **Figure 7B**, blue boxed region, see Discussion).

We next injected TH-Cre mice with AAV1-Flex-GFP at P2 and implanted cranial windows 6 weeks later. We performed *in vivo* 2-photon time-lapse imaging at 1 h intervals for 3 h. This enabled individual dendritic branches to be resolved and tracked over time, with no discernable photo-bleaching during the 3 h imaging session (**Figure 7C**). To test whether BReVI can be combined with transgenic and knock-in lines to facilitate labeling of multiple components of the glomerular circuit, we injected P2 OMP-GFP^+/-^;Thy1-YFP-G^+/-^;TH-Cre^+/-^ mice with AAV1-Flex-tdTomato. Eight weeks post-injection, we observed strong expression of tdTomato in dopaminergic neurons in the GL, in addition to GFP-expressing olfactory sensory neuron axons and YFP-expressing mitral cells arising from knock-in/transgene-mediated expression (**Figure 7D**). Hence, we labeled three important components of the OB circuit with spectrally distinct fluorescent proteins, while circumventing the extra generation of breeding (and associated time and expense) required for introduction of a fourth transgene.

### BReVI-mediated Population Labeling of Neocortical Neurons

**FIGURE 8:**
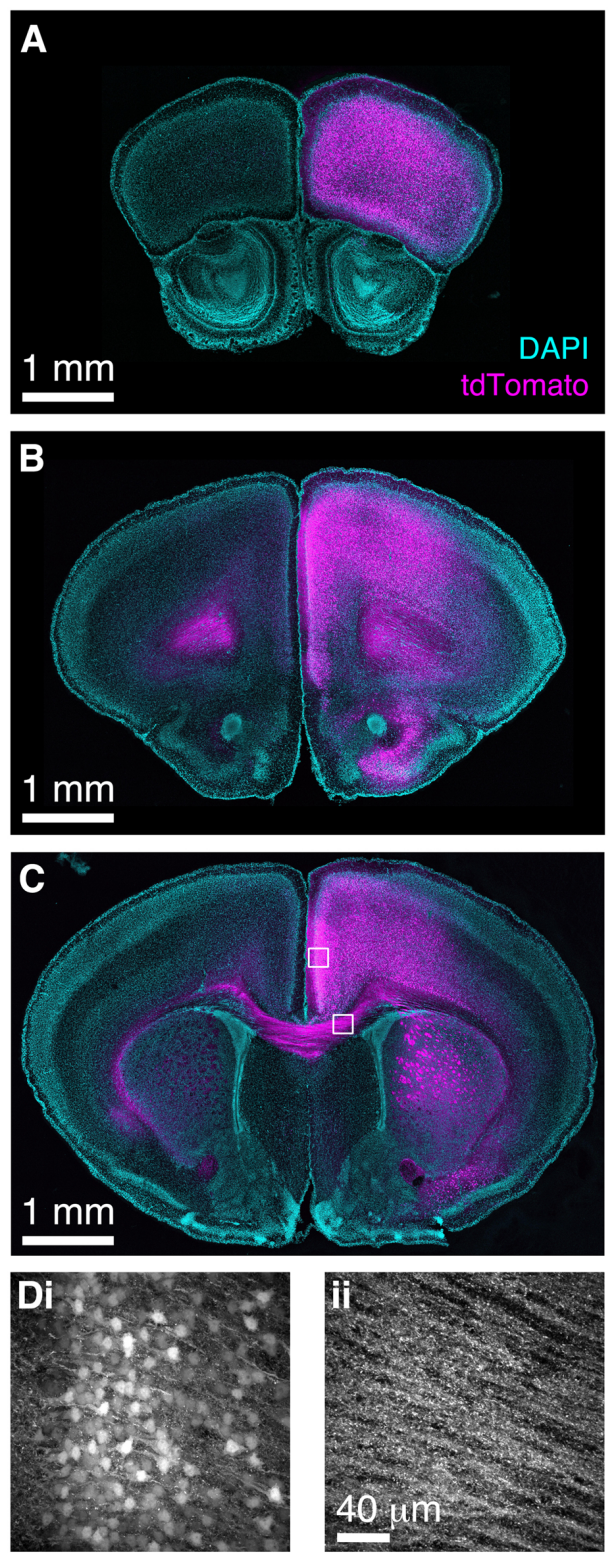
**Bulk Regional Viral Injection in frontal cortex permits population labeling of cortical CCK+ neurons and their projections. (A–C)** Coronal sections from a CCK-IRES-Cre mouse injected with AAV-Flex-tdTomato in the frontal cortex at P0. **(A–C)** are at progressively more posterior positions along the rostrocaudal axis. **(D)** MIPs of higher magnification confocal z-stacks showing tdTomato labeling of **(i)** neurons in anterior cingulate cortex and **(ii)** axons in the corpus callosum.

Finally, we wished to determine whether BReVI has broader applicability for labeling neuronal populations in other brain regions. The functional roles of genetically defined sub-populations of neocortical GABAergic interneurons are currently under intensive study (Lovett-Barron and Losonczy, 2014). CCK+ GABAergic interneurons are found throughout neocortex (Hendry et al., 1984; Lein et al., 2006; Allen Mouse Brain Atlas, 2015), but relatively little is known about their function (Galarreta et al., 2004; Xu and Callaway, 2009). To label these neurons, we injected 1 μl of AAV1-Flex-tdTomato in the right medial frontal cortex of CCK-IRES-Cre mice. Seven days post-injection, we observed strong tdTomato labeling in somata in the right frontal pole (**Figure 8A**), anterior cingulate, prelimbic, infralimbic, and motor cortex (**Figures 8B–D**). A small number of somata in the same regions of the left hemisphere were also labeled. The tdTomato expression pattern matched that seen previously using ISH (Lein et al., 2006; Allen Mouse Brain Atlas, 2015). Furthermore, we observed strong bilateral labeling of callosal fibers, including putative callosal inputs to the striatum that were stronger in the right (injected) hemisphere (**Figures 8C,D**), consistent with previous reports of CCK immunoreactivity in the corpus callosum and striatum (Meyer and Protopapas, 1985). Therefore, the CCK-IRES-Cre line, in combination with neonatal BReVI, provides an excellent tool for investigation of this poorly understood type of cortical interneurons. Furthermore, these data suggest that BReVI is a robust method for labeling of genetically defined populations of neurons in any accessible brain region.

## Discussion

### Labeling of Neuronal Populations throughout A Targeted Brain Region using BReVI

We have developed BReVI, a simple, cost-effective strategy for reporter gene expression in large numbers of neurons throughout a particular brain region. BReVI requires minimal equipment, is quick to perform (∼30 min per litter), and all injected pups survived the procedure with no evidence of injection site damage. In the OB, which comprises radial layers that enabled us to assess the spread of viral infection in three dimensions, neurons were labeled throughout the entire injected OB. Indeed, reporter gene expression appeared to be uniform in Cre-expressing neurons for an ∼2 mm radius around the injection site, in both the OB and in neocortex. Injections could be performed up to P3, and we observed strong reporter expression as early as 3 days post-injection, providing the opportunity to label and manipulate specific neuronal types both for developmental studies and for slice electrophysiology experiments, which typically use juvenile mice. Furthermore, expression was strong enough to permit *in vivo* structural and functional imaging of axons and dendrites. Hence, BReVI in neonatal Cre driver mice provides a viable alternative to, and several advantages over, genetic crosses with Cre reporter strains. First, it saves the cost of purchase, maintenance and breeding of Cre reporter strains. This may be particularly important for experiments requiring simultaneous labeling and/or manipulation of multiple neuronal types. Second, it enables new experimental tools to be adopted more rapidly; Cre reporter AAVs are readily available at low cost from commercial vector cores. Third, it may be advantageous to avoid systemic Cre-mediated recombination: for example, breeding CCK-IRES-Cre mice with a Cre-dependent diphtheria toxin line would result in ablation not only of OB tufted cells, but also CCK+ neurons in numerous other brain regions, and CCKergic endocrine cells in the small intestine, leading to unwanted side effects. Finally, for some Cre driver lines, BReVI may ameliorate ectopic reporter expression that arises from constitutive Cre expression during embryonic development (Martens et al., 2010; Lewis et al., 2013). One such example is OB granule cells, which are known to transiently express tyrosine hydroxylase mRNA, but not protein, during development (Saino-Saito et al., 2004). We found that the density of reporter-expressing neurons in the GCL was much lower in TH-Cre mice injected neonatally with AAV1-Flex-GFP than in those crossed with the ROSA-LSL-tdTomato Cre reporter line (**Figures 7A,B**). It is possible that granule cells are poorly transduced by AAV1-Flex-GFP, or that cis-acting sequences prevent GFP expression from the viral construct. Alternatively, the transient transcription from the TH promoter that presumably drives reporter expression in TH-Cre;ROSA-LSL-tdTomato mice may be largely complete by birth, such that postnatal BReVI labels only a small number of GCL neurons in TH-Cre mice.

Although we predominantly tested BReVI in the OB, the results of our injections in frontal cortex (**Figure 8**) demonstrate that BReVI has broad applicability and can be used to drive reporter expression in any accessible brain region. Similarly, stereotaxic BReVI could be used to target deeper brain structures for population labeling. However, it should be noted that BReVI is complementary to existing strategies for AAV-mediated expression and is not ideal for all purposes. In circumstances where it is essential to confine reporter expression to a discrete locus, for example when assessing the function or connectivity of a spatially restricted group of neurons, then small volume stereotaxic AAV injections in adult mice would be a better experimental strategy.

### Specific Reporter Expression in OB Tufted Cells using the CCK-IRES-Cre Mouse Line

We used BReVI to evaluate the specificity of tufted cell labeling in the OBs of CCK-IRES-Cre mice. The pattern of Cre-dependent reporter expression was strikingly similar to that seen by anti-CCK immunohistochemistry (Seroogy et al., 1985), with some degree of neuronal labeling in all layers except the olfactory nerve layer. We observed numerous tdTomato-expressing neurons with somata located close to the GL/EPL border, in the sEPL and close to the EPL/MCL border, consistent with labeling of external/superficial, middle, and internal tufted cells, respectively. We also saw strong reporter expression in a population of neurons with large somata located in the MCL, as well as sparse labeling of small neurons (likely granule cells or deep short-axon cells) in the IPL and GCL, again consistent with CCK immunohistochemistry (Seroogy et al., 1985).

Given the tdTomato-labeled neurons in the MCL, does the CCK-IRES-Cre line specifically label OB tufted cells? First, it is clear that only a small subset of principal neurons in the MCL are labeled in CCK-IRES-Cre mice, therefore we believe that this line can be used to distinguish between principal neuron sub-types. Second, the distinction between mitral and internal tufted cells can be ambiguous; indeed, internal tufted cells have also been termed ‘displaced mitral cells’ (Mori et al., 1983). Classification has been based predominantly on soma location in the MCL vs. EPL (Mori et al., 1983; Ghosh et al., 2011; Igarashi et al., 2012; Burton and Urban, 2014), with lateral dendrite projection in the dEPL vs. sEPL and axonal projection patterns (Haberly and Price, 1977; Scott, 1981; Mori et al., 1983; Nagayama et al., 2010, 2014; Igarashi et al., 2012) as additional classification criteria. However, type II mitral cells project lateral dendrites in the intermediate EPL (Orona et al., 1984; Mouradian and Scott, 1988; Nagayama et al., 2014), and may form intrabulbar axon collaterals (Ghosh et al., 2011), a feature typically thought to be specific to tufted cells (Liu and Shipley, 1994; Igarashi et al., 2012). Furthermore, it has recently been shown that a substantial fraction of late-born mitral cells extend their lateral dendrites in the sEPL (Imamura and Greer, 2015), raising the possibility that mitral and tufted cells in fact form a continuum, with their location and properties determined by birthdate (Nagayama et al., 2014; Imamura and Greer, 2015). Whether there are tufted cells in the MCL, or whether a subset of mitral cells express CCK, the CCK-IRES-Cre line labels a specific genetically defined subset of OB principal neurons, with unambiguous labeling of external and middle tufted cells, and hence will be a valuable tool for structural and functional interrogation of OB circuits. Furthermore, given their close proximity to the MCL, genetic access to internal tufted cells will be invaluable in elucidating their function and disambiguating their classification relative to mitral cells.

### Developmental Changes in OB Tufted Cell Populations

We found that the number of tdTomato-labeled tufted cells in the GL, EPL, and MCL increased dramatically between P3 and P14, and then stabilized. There was a particularly sharp increase in tufted cell density between P7 and P14; overall, the number of tdTomato-labeled neurons increased 3.8-fold over the course of the second postnatal week. This finding is consistent with a previous report that external tufted cell numbers increase during postnatal development (Marks et al., 2006), ruling out the possibility that the changes in tufted cell numbers arose from a delay in virally mediated expression. However, the source of these postnatally appearing neurons is unclear. Tufted cells are generated embryonically in the ventricular zone, with peak levels of neurogenesis at E15, and reach their final position in the OB in an inside-out sequence (Hinds, 1972; Blanchart et al., 2006; Imamura et al., 2011). Hence, it is conceivable that migration of at least the more superficially located tufted cells continues into early postnatal life. However, late migration is unlikely to account for the large increase in tufted cell numbers during the second postnatal week. Indeed, our viral labeling strategy suggests an alternative possibility: that the neurons are already present in the OB, but that CCK expression onset is delayed. AAV1 is cleared within hours of injection (van Gestel et al., 2014), but once transduced, viral DNA is episomally maintained (Penaud-Budloo et al., 2008). Hence, neurons that later undergo Cre-mediated recombination to express tdTomato must have been present in the OB at the time of injection (P0 for these experiments) in order to undergo viral transduction. Therefore, we propose that a full complement of OB principal neurons is present in the OB around birth, but that many tufted cells do not acquire their CCKergic phenotype until P14. This would have functional consequences for odor processing: CCK release by intrabulbar-projecting tufted cell axons is thought to co-ordinate the activity of isofunctional odor columns on opposite sides of the OB, although whether this is achieved by granule cell excitation or direct activation of mitral cells has been debated (Liu and Shipley, 1994; Ma et al., 2013). Furthermore, initially diffuse intrabulbar projections at P14 refine to maturity by 7 weeks of age (Marks et al., 2006). Hence, the dramatic increase in CCK expression in tufted cells is temporally matched with the onset of this refinement process, suggesting that neuropeptide release from tufted cell axon terminals may play a role in achieving the mature one-to-one specificity between tufted cells in one hemibulb, and their topographically related targets in the other.

## Author Contributions

CC designed experiments, acquired, analyzed, and interpreted data and wrote the manuscript. BG acquired data. LB contributed to experimental design. All authors critically revised the work for intellectual content.

## Conflict of Interest Statement

The authors declare that the research was conducted in the absence of any commercial or financial relationships that could be construed as a potential conflict of interest.
